# Effects of organized physical activity on quality of life and Phantom pain among adults with lower limb amputation: a systematic review and meta-analysis

**DOI:** 10.1007/s11136-025-04149-w

**Published:** 2026-01-03

**Authors:** Anastasiia Nahorna, Heiner Baur, Viktoriia Kyrychenko, Olena Andrieieva, Olena Lazarieva

**Affiliations:** 1 Department of Physiotherapy, Bern University of Applied Sciences, School of Health Professions, Bern, Switzerland; 2 Faculty of Physical Education and Sport, Babeș-Bolyai University, Cluj-Napoca Napoca, Romania; 3 Department of Fitness and Recreation, National University of Ukraine On Physical Education and Sport, Kiev, Ukraine; 4 Department of Physical Therapy and Occupational Therapy, National University of Ukraine On Physical Education and Sport, Kiev, Ukraine

**Keywords:** Amputation, Phantom pain, Rehabilitation, Quality of life, Physical activity, Meta-analysis

## Abstract

**Purpose:**

While some studies report improved outcomes following exercise-based interventions, the overall findings regarding their impact on individuals with lower limb amputation (LLA) remain inconsistent. Therefore, there is a need to synthesize the available evidence. In this regard, this systematic review and meta-analysis aimed to clarify the effects of organized physical activity on quality of life (QoL) and phantom limb pain (PLP) in adults with LLA.

**Methods:**

This review was conducted in accordance with PRISMA 2020 guidelines and registered in PROSPERO (CRD42024582703). Eight databases were searched in November 2024, including PubMed, Embase, Cochrane Library, Web of Science, PsycINFO, SPORTDiscus, ICI World of Papers, and ClinicalTrials.gov. Search terms combined concepts related to amputation, physical activity, quality of life, and phantom limb pain. Studies were included if they examined adults aged 18–60 with lower limb amputation and reported outcomes on quality of life or phantom limb pain following structured physical activity interventions. Only studies using validated outcome measures were considered.

**Results:**

Six studies met the inclusion criteria for the systematic review. Three studies (reporting four independent effect sizes) were included in the meta-analysis of QoL outcomes. The pooled effect size was small and not statistically significant (SMD = –0.06, 95% CI: –0.29 to 0.16; p = 0.58), with no observed heterogeneity (I^2^ = 0%). Narrative synthesis indicated that most studies found either non-significant or short-term improvements in QoL. Only one study reported a reduction in phantom limb pain (PLP; NPRS: 9 to 2), precluding a meta-analysis for this outcome due to insufficient data.

**Conclusions:**

Current evidence does not provide statistically significant support for improvements in QoL following organized physical activity among adults with lower-limb amputation, while effects on PLP remain unclear due to limited and heterogeneous data. Further high-quality research using standardized, context-sensitive outcome measures and well-defined intervention protocols is needed. A more person-centered and contextually grounded understanding of QoL may help capture meaningful recovery outcomes in this population.

## Background

Lower limb amputation (LLA) is a life-altering event with profound consequences for an individual's physical, psychological, and social functioning. Among the most commonly reported consequences are a reduced quality of life (QoL) and the presence of phantom limb pain (PLP), both of which can significantly impair long-term rehabilitation outcomes and daily functioning [1–4]. Given these challenges, rehabilitation programs are increasingly emphasizing structured and patient-centered interventions to support individuals with LLA in regaining autonomy and reintegrating into society.

Organized physical activity – defined as structured, repetitive, and purposeful bodily movement delivered in supervised, semi-supervised, or home-based settings [5, 6] – has shown promise in supporting physical recovery, enhancing psychosocial well-being, and reducing health-related risks in various populations. In the context of limb loss, it may contribute not only to improved mobility and functional status [7, 8], but also to reductions in secondary complications such as chronic pain, depression, and cardiometabolic disorders [8, 9]. Despite these potential benefits, the specific effects of organized physical activity on QoL and PLP in individuals with LLA remain insufficiently examined.

Previous studies have provided encouraging but inconsistent findings [10–14]. Some have reported positive outcomes following tailored exercise programs, adapted sports participation, or multidisciplinary physical therapy [15–17], while others have failed to demonstrate meaningful improvements. [9, 11, 18, 19]

The present systematic review and meta-analysis aims to address these gaps by synthesizing available evidence on the impact of organized physical activity on QoL and PLP among adults with LLA. In doing so, it seeks to identify patterns in intervention characteristics, evaluate methodological limitations, and provide evidence-informed recommendations for clinical practice and future research.

## Methods

### Protocol registration

This systematic review and meta-analysis was conducted in accordance with a pre-specified protocol registered in the PROSPERO database (PROSPERO 2024 CRD42024582703). The protocol was developed in line with the PRISMA 2020 guidelines and guided all methodological procedures.

### Eligibility criteria

Studies were eligible for inclusion if they investigated adult participants (aged 18–60 years) with lower limb amputation (LLA), defined as the partial or complete loss of a lower extremity at or below the hip joint (including transfemoral, transtibial, or partial foot levels), regardless of etiology or prosthesis use, and reported outcomes related to QoL and/or PLP. Only studies examining the effects of organized physical activity were included. Interventions could be delivered in supervised, semi-supervised, or home-based settings, and included physiotherapy, functional training, and adapted sports. Studies involving upper limb amputation, hip disarticulation, or multiple limb loss were excluded.

In line with multidimensional models of QoL [20, 21], this review focused on health-related quality of life as the primary outcome, defined as the perceived impact of health status and functional capacity on overall well-being. The concept was operationalized through validated questionnaires assessing overall QoL, acknowledging that these instruments cover partly different domains such as physical functioning, emotional well-being, pain, vitality, and social participation. Each of these instruments is grounded in established conceptual models of health-related quality of life: for instance, the Short Form Health Survey (SF-36) and RAND 36-Item Health Survey (RAND-36) are based on a multidimensional health framework encompassing physical, mental, and social functioning; the EuroQol 5-Dimension questionnaire (EQ-5D), including its 3-Level version (EQ-5D-3L) and Visual Analogue Scale component (EQ-5D VAS), is rooted in the health utility model focusing on functional health states and self-rated overall health; and the Trinity Amputation and Prosthesis Experience Scales (TAPES) and Prosthesis Evaluation Questionnaire (PEQ) were specifically developed for amputee populations to capture prosthesis-related adjustment and psychosocial adaptation.

Despite this variation, all measurement tools identified in the included studies – such as the SF-36, EQ-5D (including EQ-5D-3L and EQ-5D VAS), RAND-36, TAPES, and PEQ – are recognized measures of QoL in rehabilitation research. Therefore, studies were not restricted to specific questionnaires, and total or summary QoL scores were used to ensure comparability across studies.

The primary outcomes were changes in QoL and PLP. For QoL, only total or summary scores from validated questionnaires were extracted and analyzed, without considering individual domains separately. PLP was assessed using validated pain questionnaires, most commonly the McGill Pain Questionnaire (MPQ), Brief Pain Inventory (BPI), and Visual Analogue Scale (VAS), although other standardized measures of pain intensity or interference were also eligible for inclusion, provided they demonstrated validity and reliability.Studies were excluded if they:

- Focused on children, adolescents (< 18 years), or older adults (> 60 years),
- Used mirror therapy or mental imagery as the sole intervention [22–24],
- Implemented passive or non-volitional activities (e.g., electrical stimulation without movement),
- Did not report outcomes on QoL or PLP using validated measurement tools.

Eligible study designs included randomized controlled trials (RCTs), non-randomized controlled trials, case–control studies, and cross-sectional studies (including pre-post single group designs). There were no restrictions on language, publication year, or publication status.

### Search strategy

A comprehensive search strategy was developed in accordance with PRISMA guidelines and applied to the following databases: PubMed, Embase, Cochrane Library, Web of Science, PsycINFO, SPORTDiscus, ICI World of Papers, and ClinicalTrials.gov. Searches were conducted throughout November 2024 and completed on November 22, 2024.

No language, dates or publication status restrictions will be used. No restriction by race or sex.

Search terms were based on four core concepts: amputation, physical activity, quality of life, and phantom limb pain. The strategy was developed in collaboration with a medical librarian experienced in systematic reviews. Controlled vocabulary (e.g., MeSH) and free-text terms were combined using Boolean operators. A sample search strategy for PubMed, which was subsequently adapted for other databases, is presented in Appendix A.

### Selection of sources of evidence

All references retrieved from the database searches (n = 1,888) were initially imported into Citavi for organization and duplicate removal. A total of 888 duplicates were identified and removed. The remaining 1,000 unique records were uploaded into Rayyan, an online platform for systematic reviews, to facilitate independent screening.

Two reviewers (A.N., V.K.) independently screened the titles and abstracts based on predefined eligibility criteria. At the time of writing, a subset of references had been marked as resolved or excluded, with no unresolved conflicts remaining. Any disagreements were resolved through discussion or by consulting a third reviewer (H.B.).

Full-text assessment was subsequently conducted by the same reviewers, and reasons for exclusion were documented.

### Data extraction

Two reviewers (A.N. and V.K.) independently extracted data and cross-checked each other’s entries to ensure consistency and completeness. Any disagreements were resolved through discussion or, if necessary, with input from a third reviewer (H.B.). Data extraction followed a structured template that had been piloted in advance and adapted to the scope of the review. The form captured key study characteristics (e.g., authorship, publication year, country, and study design), participant demographics (including age, sex, and amputation level), specifics of the physical activity intervention (type, frequency and duration), and outcome-related information (measurement tools for QoL and PLP, time points, and numerical results). The decision regarding whether a study met the criteria for inclusion in the meta-analysis was made by the first author (A.N.) based on the completeness and compatibility of the reported data. Data extraction was performed using Microsoft Excel (version 16.72), with all entries organized and stored in structured spreadsheets developed for this review. All extracted data were summarized in structured tables.

### Risk of bias assessment

The methodological quality and risk of bias of the included studies were assessed using tools appropriate for each study design. For the randomized controlled trial (RCT), the Cochrane Risk of Bias 2.0 (RoB 2.0) [25, 26] tool was applied, while for non-randomized studies – including pre-post single group designs and case reports – the Downs and Black checklist [27] was used. Detailed domain structures and scoring criteria for both instruments are provided in Supplementary Appendices B and C. All assessments were conducted independently by two reviewers (A.N. and V.K.), with disagreements resolved through discussion or by consulting a third reviewer (H.B.).

According to the Cochrane Handbook for Systematic Reviews of Interventions [25], RoB 2.0 assesses bias across five domains and assigns an overall judgment for each study as “low risk of bias,” “some concerns,” or “high risk of bias” and an overall risk of bias judgment is derived using an algorithm: low risk of bias – all domains are rated as low risk; some concerns – at least one domain is rated as “some concerns,” but none are rated as “high risk”, high risk of bias – one or more domains are rated as high risk, or there are multiple domains with “some concerns” that substantially lower confidence in the result [25].

For the Downs and Black checklist, the total score was calculated based on the original version comprising 27 items [27, 28]. As there is no universally accepted cut-off for interpreting overall methodological quality, we applied a classification scheme commonly used in previous systematic reviews: low risk of bias – 26–27 points; moderate risk of bias – 20–25 points; high risk of bias – < 20 points. These thresholds were pre-specified prior to data extraction to ensure consistency and transparency in interpretation.

### Synthesis of findings

A total of six studies were included in the systematic review. [9, 15–19] All were assessed for outcomes related to both QoL and PLP. Among them, three studies reported sufficient quantitative data (including means and standard deviations) for QoL outcomes using validated instruments and were included in the meta-analysis. A random-effects model was applied to calculate standardized mean differences (SMD) for these studies.

The remaining three studies [15, 16, 18] were excluded from the meta-analysis because essential statistical data were missing or QoL was reported using non-standardized, study-specific approaches (e.g., narrative descriptions or non-validated checklists), which prevented quantitative synthesis. However, they were reviewed narratively and included in the descriptive synthesis to ensure that relevant trends and intervention characteristics were still captured.

All six studies were also assessed for their reporting on phantom limb pain. Although PLP was addressed in several studies, the diversity of measurement instruments, inconsistent reporting of results, and lack of usable statistical data (e.g., mean ± SD or change scores) prevented pooled analysis.

The criteria for inclusion in the meta-analysis were: (1) structured physical activity as the main intervention, (2) adult participants with major lower limb amputation, (3) validated QoL instruments used pre- and post-intervention, and (4) sufficient statistical information to calculate effect sizes. Intervention characteristics were extracted from all studies and compared descriptively.

## Results

### Literature search and description of the included studies

**Fig. 1:**
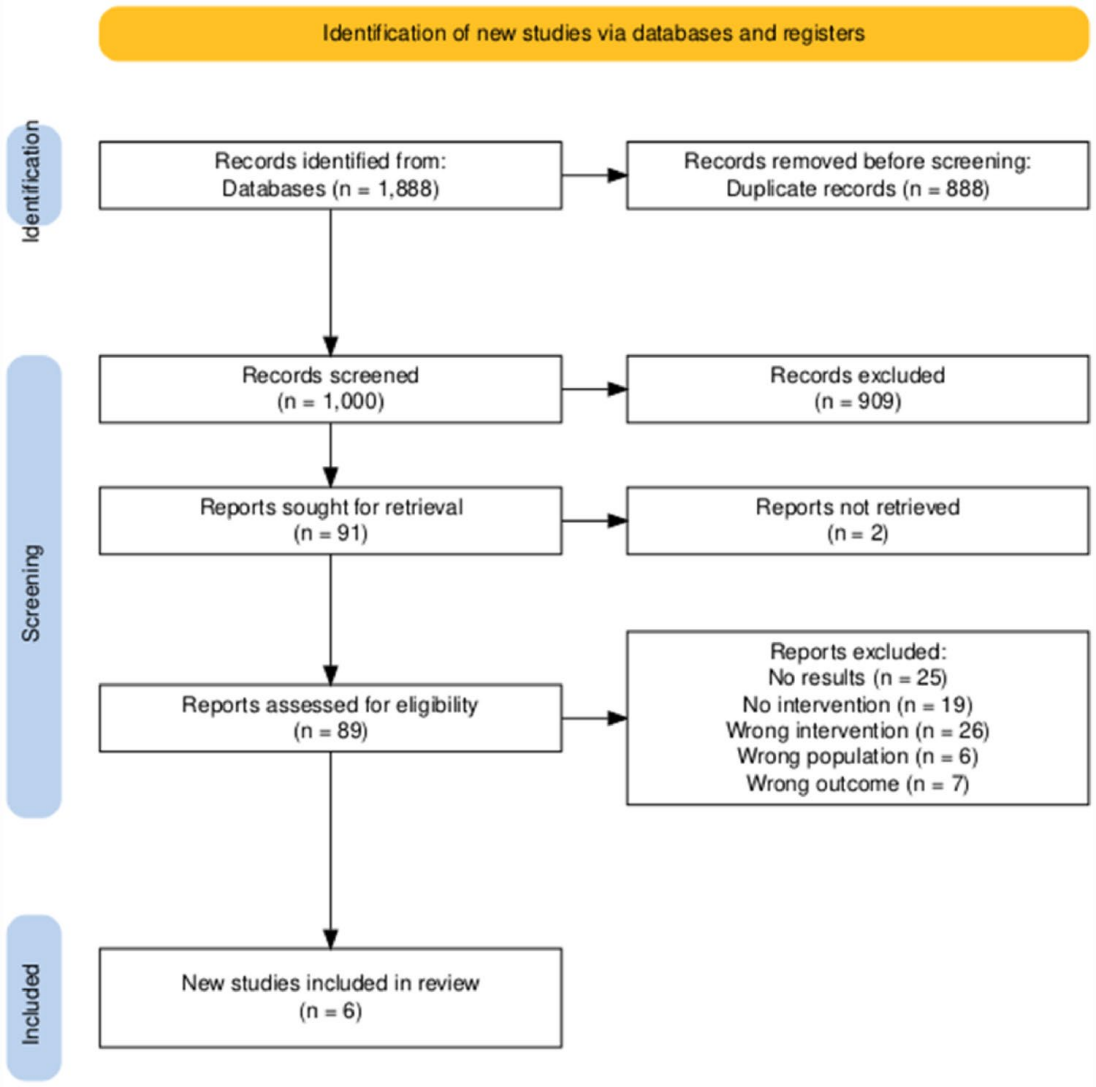
PRISMA Flow Chart

A total of 1,888 records were identified through database searching, with no additional records retrieved from other sources (Fig. 1). After removing 888 duplicates, 1,000 unique records remained for title and abstract screening. Of these, 909 were excluded based on relevance. The remaining 91 articles were sought for full-text retrieval, but 2 could not be accessed due to lack of open access. Attempts were made to contact the corresponding authors, but no responses were received. Thus, 89 articles were assessed for eligibility. Of the 89 full-text articles assessed, 83 were excluded for the following reasons: no results reported (e.g., no access to full text, unpublished, abstract only) (n = 25); no intervention (e.g., observational, correlational, reviews) (n = 19); wrong intervention (e.g., mirror therapy, mental imagery, mixed reality systems, mobility devices, passive methods, software tools) (n = 26); wrong population (e.g., outside the target age range or inappropriate amputation type) (n = 6); and use of non-validated outcome measures (e.g., no QoL or PLP, or outcomes not appropriately measured) (n = 7).

Six studies met all inclusion criteria and were included in the systematic review (Table 1). Of these, three studies were eligible for quantitative synthesis (meta-analysis of quality of life outcomes only). Studies reporting PLP outcomes varied in tools and results, and were excluded from the meta-analysis due to the lack of mean ± SD data, or because structured physical activity was not the primary variable of interest. Structured information regarding the included studies is presented in tabular format, including details on study characteristics and reasons for the exclusion of certain studies from the meta-analysis.

**Table 1:** Study Characteristics

Study	Country	Study Design	Sample Size	Age	Sex (Male/Female)	Amputation Level
Godlwana et al. [17]	South Africa	RCT	154 enrolled/ 121 completed	58	100/54	Above/below knee
Kim et al. [9]	Republic of Korea	Pre-post single group	15 enrolled/ 14 completed	46.1 ± 10.3	14/0	Above/below knee (below-ankle was excluded)
Mosteiro-Losada et al. [19]	Spain	Pre-post single group	6	56.83 ± 9.70	5/1	Above/below knee
Oorschot et al. [18]	Netherlands	Pre-post single group	13 enrolled/ 5 completed	Median 51	Not specified	Above/below knee
Rosenblatt et al. [16]	USA	Case Report	1	58	1/0	Below-knee
Sheikh et al. [15]	India	Case report	1	59	1/0	Below-knee

RCT: randomized controlled trial

The studies included in the systematic review were conducted in six countries: South Africa, Republic of Korea, Spain, the Netherlands, the United States, and India. Study designs comprised one randomized controlled trial (RCT), three pre-post single group studies, and two case reports. In total, 190 participants were enrolled across all six studies, of whom 148 completed post-intervention assessments and were included in the synthesis.

A total of 121 men and 55 women were explicitly reported. The sex of participants was not specified in one study [18]. Most studies involved adults with transtibial (below-knee) or transfemoral (above-knee) amputation. Four studies included both levels (with one9 explicitly excluding below-ankle amputation) [9, 17, 18, 20] while two focused exclusively on below-knee amputation [15, 16].

The average age of participants across the included studies was approximately 54.8 years; however, this value should be interpreted with caution due to variability in reporting. Some studies provided only median age, omitted standard deviations, or reported age as a range, limiting the precision of overall age estimates.

The studies included in the meta-analysis were conducted in South Africa, the Republic of Korea, and Spain. They consisted of one RCT and two pre-post single group studies. A total of 175 participants were enrolled in these studies, with 141 completing post-intervention assessments and included in the quantitative synthesis. The average age was estimated at 53.6 years, although precision is limited due to inconsistent reporting formats (e.g., mean ± SD vs. median or reported age as a range). All included studies involved participants with major lower limb amputation (i.e., transtibial or transfemoral), which precluded comparisons across more specific amputation levels.

## Summary of the risk of bias assessment

Table 2 presents the methodological quality ratings for each included study, assessed using tools appropriate for the respective study designs. For the randomized controlled trial (RCT), the Cochrane Risk of Bias 2.0 (RoB 2.0) tool was applied, while for non-randomized studies – including pre-post single group designs and case reports – the Downs and Black checklist was used.

**Table 2:** Summary of the risk of bias assessment

Study	Study Design	Score	Quality
Godlwana et al. [17]	RCT	–	Low
Kim et al. [9]	Pre-post single group	13	High risk of bias
Mosteiro-Losada et al. [19]	Pre-post single group	15	High risk of bias
van Oorschot et al. [18]	Pre-post single group	22	Moderate
Rosenblatt et al. [16]	Case report	13	High risk of bias
Sheikh et al. [15]	Case report	12	High risk of bias

RCT: randomized controlled trial; RoB 2.0: Cochrane Risk of Bias tool; modified Downs and Black checklist used for non-randomized studies

The RCT [17] was judged to have a low risk of bias based on the RoB 2.0 criteria. For non-randomized studies, scores on the modified Downs and Black checklist were used to classify overall quality. Based on these, four studies were classified as having high risk of bias, one as moderate, and none as low. Full details are presented in Table 2.

Studies rated as having a high risk of bias most frequently showed methodological limitations related to participant selection, lack of control or blinding, and incomplete outcome data.

### Intervention characteristics and outcomes measurements

Table 3 summarizes the characteristics of exercise-based interventions and outcome measures across the six included studies. The interventions demonstrated substantial variation in structure, delivery mode, and exercise prescription parameters (type, frequency, duration, and intensity). Programs ranged from home-based exercise and digital health interventions to supervised physiotherapy and structured group-based sports or multimodal programs integrating physical training with cognitive-behavioral therapy or virtual reality.

**Table 3:** Intervention Characteristics and Outcomes Measurements

Study	Intervention Type	Duration/ Session Duration	Intensity / Format	Frequency	QoL Tool	Phantom Pain Tool
Godlwana et al. [17]	Home-based exercise and education (stretching, strengthening, balance and mobility training, transfer practice, lifestyle education)	24 weeks/NR	Not explicitly reported; inferred as low–moderate based on content (stretching, balance, functional training, home-based daily program).; self-administered at home with illustrated handouts, telephonic reminders, and exercise diary	Daily	EuroQoL-5D (VAS and Index)	Not measured
Kim et al. [9]	Digital healthcare management program: home-based self-directed physical-strength and cardiovascular exercise (warm-up, stretching, muscle strengthening, balance and stability training, cool-down)	12 weeks/ ~ 60 min/session (30 min strength + 30 min walk)	Not explicitly reported; prescribed intensity: 60–80% HRmax for cardiovascular component; strength training with graded difficulty (easy–hard); self-directed via mobile app, without direct monitoring	3 per week	EQ-5D-3L	Not measured
Mosteiro-Losada et al. [19]	Tailored core stability and balance circuit training (breathing, body awareness, strengthening, stretching, and mobility exercises using resistance bands and light weights)	20 weeks/ 1 h (weeks 1–2); 2 h (weeks 3–20)	Progressive intensity (initial low → moderate); supervised circuit training with individualized load adjustments (1–2 kg increments every 2 weeks); adapted for fitness level and amputation type	1 per week	SF-36	Not measured
Oorschot et al. [18]	Group-based fitness, swimming, and adapted sports training guided by sports therapists; initial 6-week intensive phase (strength and cardiovascular training) followed by 8-month maintenance phase focusing on participation in various parasports (soccer, sitting volleyball, wheelchair basketball, boxing, golf). Participants were medically screened by a sports physician and prosthetist before starting	6 weeks (3/week), then weekly for 8 months/NR	Not explicitly reported; inferred as moderate to vigorous, combining strength and endurance components; progression from structured fitness to sports participation	6 weeks (3 per week), then 1 per week for 8 months	RAND-36	Not measured
Rosenblatt et al. [16]	Integrating virtual reality program –based active gaming (physical therapy component) with CBT strategies targeting balance confidence, avoidance behaviors, and maladaptive cognitions. Sessions guided by a physical therapist and behavioral counselor. Control group performed seated upper-body exercises at home	8 weeks/90 min	Not explicitly reported; inferred as moderate, involving active movement through VR gaming	1 per week	PEQ	Not measured
Sheikh et al. [15]	Postoperative exercise therapy – respiratory and range of motion (ROM) exercises, strengthening, balance and gait training, posture correction, and pre-prosthetic gait preparation performed under physiotherapist supervision	Not clearly specified (initiated on postoperative day 2 and continued until functional independence)/ 15–20 min per component 2–3 times daily	Not explicitly reported; inferred as low to moderate, progressive according to recovery stage	2–3 times daily for most components	Not measured	NPRS

QoL: quality of life; PLP: phantom limb pain; NR: not reported; NPRS: Numeric Pain Rating Scale; SF-36, EQ-5D, EQ-5D-3L, TAPES, and PEQ – validated health-related quality of life questionnaires

The duration of interventions varied from 6 to 24 weeks, except for the postoperative program by Sheikh et al. [15], which began on postoperative day 2 and continued daily until functional independence without a predefined endpoint. Frequency ranged from daily sessions [15, 17] to 2–3 sessions per week [9, 18], or once weekly [16, 19]. Session duration ranged between 15–20 [15] minutes per exercise component in early rehabilitation and up to 90–120 min in structured fitness or training programs [9. 16]. Intensity was generally low to moderate in early rehabilitation studies and progressively increased to moderate or vigorous levels in structured training and sports interventions. Quality of life (QoL) outcomes were assessed using validated instruments, including EuroQol-5D, EQ-5D-3L, SF-36, RAND-36, and PEQ.

Most studies described improvements in QoL measures following intervention, though the magnitude and consistency of change varied across studies. Phantom limb pain (PLP) was assessed in one study (Sheikh et al., 2024) using the Numeric Pain Rating Scale (NPRS) [15]. Other studies primarily focused on QoL outcomes, with Oorschot et al. [18] reporting functional improvements in mobility without corresponding QoL changes, and Rosenblatt et al. [16] noting temporary gains in PEQ scores that returned to baseline at follow-up.

### Evaluation of claimed effectiveness of organized physical activity on quality of life and phantom pain

**Fig. 2:**
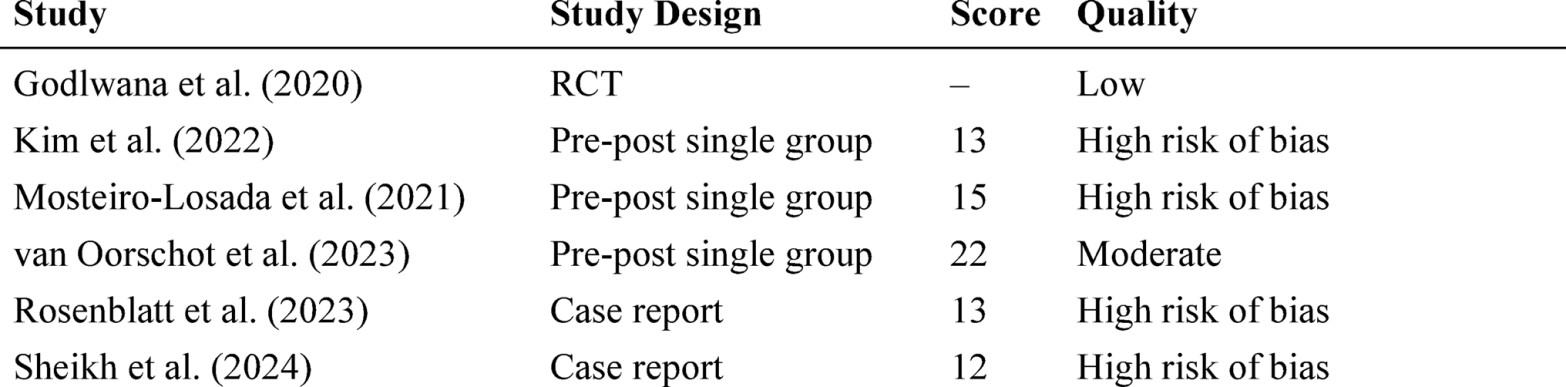
Summary of QoL and PLP Assessment Results in Included Studies. *Note: QoL – quality of life; PLP – phantom limb pain; ↑ improvement; ↓ decrease; ↔ no change*

As illustrated in the accompanying Fig. 2 summarizing the direction of changes in QoL and PLP outcomes across studies, Godlwana et al. reported significant improvements in EQ-5D VAS scores at both 3 and 6 months (p = 0.003; p = 0.033), although no statistically significant change was observed in the EQ-5D Index (p = 0.25)[17]. Kim et al. and Mosteiro-Losada et al. reported slight increases in QoL scores (EQ-5D-3L and SF-36 respectively), but these improvements did not reach statistical significance [9, 19]. Oorschot et al. observed functional gains in mobility and walking capacity, though no meaningful changes were reported in RAND-36 QoL domains [18]. In the study by Rosenblatt et al. temporary improvements in PEQ scores were noted after four sessions, but these gains regressed to baseline levels at follow-up [16]. While PLP outcomes were not assessed in most studies, the case report by Sheikh et al. documented a notable reduction in phantom limb pain, with NPRS scores decreasing from 9 to 2, alongside improvements in range of motion and muscle strength [15].

### Meta-analysis of effectiveness of organized physical activity on quality of life

**Fig. 3:**
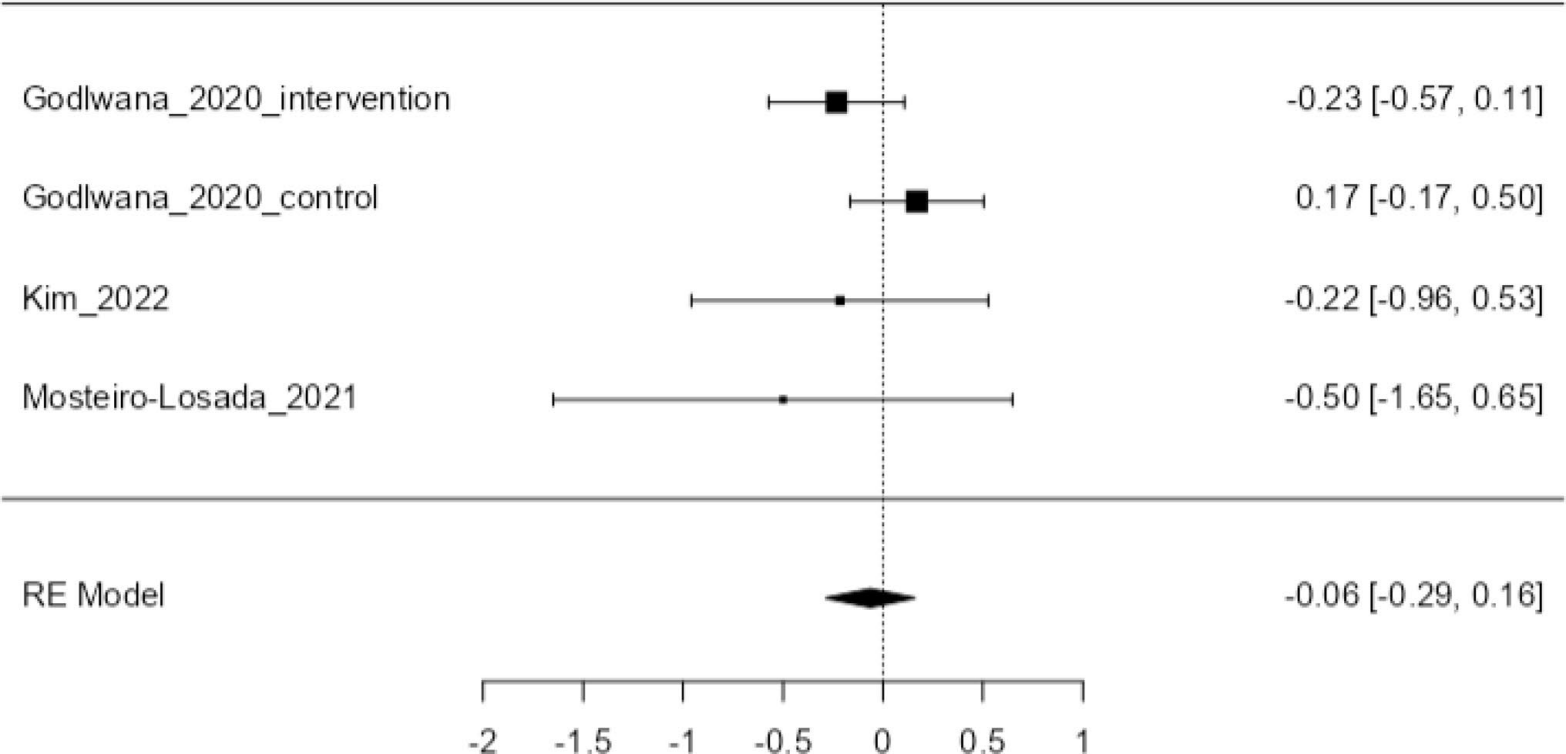
Meta-analysis of effectiveness of organized physical activity on quality of life. *Note: Squares represent individual study effects; diamond indicates pooled estimate (random-effects model)*

A meta-analysis was conducted to evaluate the effects of interventions on QoL among individuals with lower-limb amputation, using standardized mean differences (SMD) as the outcome metric [29] (Fig. 3). The analysis included three studies, one of which contributed separate data from both an intervention and a control group, resulting in four effect sizes (k = 4). A random-effects model was applied [30].

Given the heterogeneity of QoL instruments used across studies, SMDs were used to enable pooling of outcomes measured on different scales. All included tools assessed health-related quality of life (HRQoL), although they differ in their conceptual underpinnings. Instruments such as the SF-36 and EQ-5D Visual Analogue Scale (VAS) reflect direct self-rated health perceptions, while the EQ-5D-3L Index represents a utility-based health score derived from weighted domain values. When both Index and VAS values were reported, only the VAS data were used to maintain conceptual consistency across studies. However, when only EQ-5D-3L Index data were available, these were included to avoid unnecessary data loss and preserve completeness of evidence, acknowledging the potential conceptual heterogeneity this may introduce.

The pooled effect estimate was non-significant, with an average SMD of –0.0629 (95% CI: –0.2859 to 0.1601, z = –0.553, p = 0.580), indicating no significant change in QoL across the included studies. Heterogeneity was negligible, as indicated by τ^2^ = 0, I^2^ = 0%, and a non-significant Cochran’s Q-test (Q(3) = 3.53, p = 0.317), suggesting consistent effects across studies.

Figure 3 illustrates the individual effect sizes with their 95% confidence intervals, as well as the pooled estimate at the bottom of the plot, represented by a diamond shape.

## Discussion & limitations

This systematic review and meta-analysis evaluated the available evidence on the effects of organized physical activity on QoL and PLP in adults with lower-limb amputation. While structured physical activity interventions are widely advocated for enhancing physical and psychological recovery, the findings revealed a small, statistically non-significant effect on QoL. The pooled estimate from the meta-analysis (SMD = –0.0629; 95% CI: –0.2859 to 0.1601; p = 0.580) demonstrated no meaningful improvement, and heterogeneity was negligible (I^2^ = 0%), indicating consistent findings across studies.

The narrative synthesis reflected a similar trend. Only one study [17] reported a statistically significant improvement in EQ-5D VAS scores, while the EQ-5D Index showed no change. Other studies [9, 16, 19] reported either modest, non-significant improvements or temporary effects that were not sustained at follow-up. One study [18] demonstrated functional gains in mobility but no associated improvement in RAND-36 QoL domains. Collectively, these findings suggest that QoL may not be directly or immediately responsive to physical performance gains, and that its measurement may depend on broader psychosocial and contextual factors.

An important challenge in interpreting these results lies in the difficulty of isolating the unique contribution of physical activity. In nearly all included studies, physical activity was delivered as part of a broader intervention package incorporating elements such as cognitive-behavioral therapy, education, digital tools, or virtual reality. Consequently, it was not possible to determine whether observed effects – positive or negative – were attributable to the movement-based component or the broader therapeutic environment. This reflects a common limitation in rehabilitation research, where multimodal interventions are both clinically relevant and methodologically complex.

Another critical consideration concerns the suitability of commonly used QoL instruments in this population. Most studies employed general health-related QoL questionnaires such as the EQ-5D, SF-36, or RAND-36, which, although validated, may lack sensitivity to capture changes meaningful to individuals with limb loss. QoL is a subjective construct that may be influenced by contextual factors, mood, and self-perception. Furthermore, these instruments assess higher-order domains such as social participation or emotional wellbeing, which may not align with the immediate concerns of individuals recovering from amputation – such as regaining independence, stability, and bodily control. This raises a broader conceptual consideration: the construct of “quality of life” as traditionally measured may not fully align with the immediate priorities of individuals recovering from major limb loss. According to Maslow’s hierarchy of needs [29], individuals must first satisfy foundational needs – such as safety, autonomy, and physical functioning – before addressing higher-level psychological or social goals. Future research should therefore consider complementing standardized QoL tools with needs-based or goal-oriented outcome measures that better reflect the lived experience and recovery trajectory of this population.

Additionally, this review excluded studies focused on mirror therapy, despite its frequent use in PLP management. This decision was conceptually justified: although mirror therapy involves movement-like interactions, it is primarily understood as a sensorimotor illusion technique [24] that produces therapeutic effects through cortical reorganization, not through structured, volitional physical activity [32]. Including such studies would have introduced methodological heterogeneity and compromised the review’s focus on physiologically active rehabilitation strategies.

Several additional limitations merit mention. The number of eligible studies was small (n = 6), and only three provided complete data for meta-analysis. Most studies were non-randomized, small-sample, and assessed as having moderate to high risk of bias. There was substantial variation in intervention formats, durations, and frequencies. Outcome measures were inconsistently applied, and long-term follow-up was largely absent. Demographic data such as age, sex, and prosthesis use were incompletely reported in some studies, limiting generalizability. A further limitation is that publication bias could not be assessed formally due to the small number of studies included in the meta-analysis, which may also restrict the generalizability of findings. Finally, the heterogeneity of QoL instruments across studies should be acknowledged. Although standardized mean differences (SMDs) were applied to enhance comparability, conceptual differences between self-rated (e.g., SF-36, EQ-5D VAS) and utility-based (e.g., EQ-5D-3L Index) measures may have introduced minor conceptual heterogeneity. Overall, the strength of the evidence should be regarded as low to moderate due to methodological limitations and small sample sizes across studies.

## Conclusion

In summary, this systematic review found no statistically significant evidence that organized physical activity improves quality of QoL in adults with LLA. However, this finding likely reflects methodological limitations, small sample sizes, and the use of outcome measures that may not fully capture patient priorities. Evidence regarding the effects of physical activity on PLP remains limited and inconsistent, largely due to heterogeneous interventions and insufficient statistical reporting.

These results highlight the need for rigorously designed studies with clearly defined intervention components, standardized yet context-sensitive outcome tools, and adequately powered samples. Future research should adopt a more person-centered approach to evaluating recovery, incorporating measures that reflect both functional progress and lived experience. A refined conceptualization of QoL – one that integrates the physical, psychological, and social dimensions most relevant to individuals with limb loss – may be essential to guide future research and rehabilitation practice.

## Data Availability

All data generated or analyzed during this study are included in this published article and its supplementary files. Additional details are available from the corresponding author upon reasonable request.

## Appendix A: PubMed Search Strategy – Structured Table

**Table:**

Search Number	Query
15	#4 AND #8 AND #14
14	#9 OR #10 OR #11 OR #12 OR #13
13	"mental health"[tiab] OR "emotional well-being"[tiab] OR "psychological health"[tiab]
12	"phantom limb pain"[tiab] OR "phantom pain"[tiab] OR "pain management"[tiab] OR "pain reduction"[tiab] OR "chronic pain"[tiab] OR "pain relief"[tiab]
11	"Phantom limb"[mh] OR "Pain Management"[mh]
10	"quality of life"[tiab] OR QoL[tiab] OR "life satisfaction"[tiab] OR "well-being"[tiab] OR "SF-36" OR "Short Form 36"[tiab]
9	"Quality of Life"[mh] OR "Pain Measurement"[mh]
8	#5 OR #6 OR #7
7	"strength training"[tiab] OR "aerobic exercise"[tiab] OR "endurance training"[tiab] OR "therapeutic exercise"[tiab]
6	"physical activity"[tiab] OR "exercise"[tiab] OR "fitness training"[tiab] OR "rehabilitation"[tiab] OR "exercise therapy"[tiab]
5	"Exercise"[MH] OR "Exercise Therapy"[MH:noexp] OR "Exercise Movement Techniques"[MH:noexp]
4	#1 OR #2 OR #3
3	"amputee"[tiab] OR "limb loss"[tiab] OR "amputation patient"[tiab] OR "prosthetic limb"[tiab]
2	"lower limb amputation"[tiab] OR "lower extremity amputation"[tiab] OR "leg amputation"[tiab] OR "foot amputation"[tiab] OR "below-knee amputation"[tiab] OR "above-knee amputation"[tiab]
1	("Amputation, Surgical"[mh:noexp] OR "Amputees"[mh] OR "Artificial Limbs"[mh]) AND "Lower Extremity"[mh]

## Appendix B: Cochrane Risk of Bias 2.0 (RoB 2.0) – Domains and Judgement Criteria

**Table:**

Domain	Description	Judgement Criteria
D1	Bias arising from the randomization process	Sequence generation, allocation concealment, baseline differences
D2	Bias due to deviations from intended interventions	Adherence, contamination, blinding of participants and personnel
D3	Bias due to missing outcome data	Attrition, reasons for missing data, handling of missing data
D4	Bias in measurement of the outcome	Blinding of outcome assessors, validity and reliability of measures
D5	Bias in selection of the reported result	Selective reporting, consistency between registered and reported outcomes

Overall judgment:

Low risk of bias – all domains low risk.

Some concerns – at least one domain with some concerns, none high.

High risk of bias – one or more domains high risk or multiple domains with some concerns.

*Adapted from: Higgins JPT**, **Thomas J**, **Chandler J**, **Cumpston M**, **Li T**, **Page MJ**, **Welch VA (eds). Cochrane Handbook *for* Systematic Reviews of Interventions, 2nd ed. Chichester (UK): John Wiley & Sons; 2019.*

## Appendix C: Downs and Black Checklist (27-item version)

**Table:**

Domain	No. of Items	Judgement Criteria
Reporting (10 items)	10–13	Clarity of study aims and hypotheses; description of main outcomes, interventions, participant characteristics, confounders, and findings; estimates of random variability; reporting of adverse events; description of actual probability values
External Validity (3 items)	11–13	Representativeness of participants, staff, and study setting to the wider population; relevance of interventions and context to routine clinical practice
Internal Validity – Bias (7 items)	14–20	Appropriateness of blinding (participants, investigators, outcome assessors); reliability and validity of outcome measures; data dredging; compliance with interventions; accuracy of outcome measurement
Internal Validity – Confounding/Selection Bias (6 items)	21–26	Randomization procedures; comparability of groups at baseline; adjustment for confounding factors; concealment of intervention assignment; recruitment from the same population; contemporaneous controls
Power (1 item)	27	Whether the study includes a formal sample size or power calculation to detect a clinically important effect

Overall judgment:

Maximum score: 27 points.

Quality interpretation (as applied in this review):

Low risk of bias: 26–27 points.

Moderate risk of bias: 20–25 points.

High risk of bias: < 20 points.

*Adapted from: Downs SH, Black N. The feasibility of creating a checklist for the assessment of the methodological quality both of randomised and non-randomised studies of health care interventions. Journal of Epidemiology & Community Health. 1998;52(6):377–384.*
